# Bone Angiogenesis and Vascular Niche Remodeling in Stress, Aging, and Diseases

**DOI:** 10.3389/fcell.2020.602269

**Published:** 2020-11-26

**Authors:** Sina Stucker, Junyu Chen, Fiona E. Watt, Anjali P. Kusumbe

**Affiliations:** ^1^Tissue and Tumor Microenvironments Group, Kennedy Institute of Rheumatology, NDORMS, University of Oxford, Oxford, United Kingdom; ^2^Department of Prosthodontics, West China Hospital of Stomatology, Sichuan University, Chengdu, China; ^3^Centre for Osteoarthritis Pathogenesis Versus Arthritis, Kennedy Institute of Rheumatology, NDORMS, University of Oxford, Oxford, United Kingdom

**Keywords:** angiogenesis, vascular niche, inflammation, bone metastasis, arthritis, bone marrow microenvironment

## Abstract

The bone marrow (BM) vascular niche microenvironments harbor stem and progenitor cells of various lineages. Bone angiogenesis is distinct and involves tissue-specific signals. The nurturing vascular niches in the BM are complex and heterogenous consisting of distinct vascular and perivascular cell types that provide crucial signals for the maintenance of stem and progenitor cells. Growing evidence suggests that the BM niche is highly sensitive to stress. Aging, inflammation and other stress factors induce changes in BM niche cells and their crosstalk with tissue cells leading to perturbed hematopoiesis, bone angiogenesis and bone formation. Defining vascular niche remodeling under stress conditions will improve our understanding of the BM vascular niche and its role in homeostasis and disease. Therefore, this review provides an overview of the current understanding of the BM vascular niches for hematopoietic stem cells and their malfunction during aging, bone loss diseases, arthritis and metastasis.

## Introduction

In the skeletal system, vasculature plays a crucial role in nutrient delivery and maintenance of the resident stem and progenitor cells that regulate osteogenesis and hematopoiesis. Bone marrow (BM) harbors stem and progenitor cells of different lineages including hematopoietic and mesenchymal stem cells that differentiate into a variety of mature functional cells, contributing to osteogenesis and hematopoiesis (Sivan et al., 2019). These stem and progenitor cells reside in specialized local microenvironments within the BM, known as BM niches (Colmone and Sipkins, 2008; Marenzana and Arnett, 2013). BM niches provide crucial signals for stem and progenitor cell survival, quiescence, mobilization, and differentiation. These signals come in the form of soluble factors, cell surface ligands or cell-to-cell interactions which regulate stem and progenitor cell fates (Colmone and Sipkins, 2008; Sugiyama and Nagasawa, 2012).

The BM microenvironment is highly sensitive to stress. Growing evidence suggests that stress-induced molecular changes of the BM microenvironment disrupt homeostasis (Batsivari et al., 2020). BM endothelial cells (ECs) and their secreted factors, called angiocrine factors, regulate hematopoietic stem and progenitor cell homeostasis and function. Stress associated with aging, inflammation, bone diseases or bone malignancies can disrupt vascular morphology and angiocrine signaling, with significant impacts on osteogenesis, bone angiogenesis, and hematopoiesis. The response of the BM microenvironment to stressful conditions and diseases has received increased attention over the past few years. Nevertheless, the knowledge about the effects of stress on the BM microenvironment remains incomplete and is a hot topic of research. This review aims to define the cellular and molecular response of the BM vascular niche to different stresses by comparing the BM vascular niches in homeostasis and under various stress conditions such as aging, inflammation and malignancy.

## Bone Marrow Vascular Niches in Health and Homeostasis

The BM harbors multiple different cells types, thus forming various local niches for stem and progenitor cells (Colmone and Sipkins, 2008; Sugiyama and Nagasawa, 2012). These niche-specific cell types show differential expression of specific cell-surface markers and intracellular proteins and have been characterized by genetic labeling and lineage tracing. Bone-forming osteoblasts were the first niche cells to be associated with hematopoiesis (Taichman and Emerson, 1994). They secrete hematopoietic cytokines to maintain hematopoietic stem cells (HSCs), providing an endosteal niche (Taichman and Emerson, 1994; Calvi et al., 2003; Visnjic et al., 2004). The endosteum shows high expression of important pro-hematopoietic factors, including C-X-C motif chemokine 12 (CXCL12) and stem cell factor (SCF) (Kinashi and Springer, 1994; Sugiyama et al., 2006; Ho and Méndez-Ferrer, 2020). However, osteoblast-specific deletion of these factors has little effect on HSCs, suggesting the existence of other niches for HSCs (Ding and Morrison, 2013). Moreover, various studies have shown that endosteal niches only contain a small proportion of HSCs (Kiel et al., 2007; Ding et al., 2012).

Bone marrow ECs play a crucial role in osteogenesis, bone angiogenesis, and hematopoiesis. Located at the interface between blood vessel lumen and the BM, they respond to various stimuli, including chemical or mechanical stimuli, and regulate cellular crosstalk between the two compartments. BM ECs are remarkably heterogeneous in cell surface protein expression and their response to stress and injury (Colmone and Sipkins, 2008; Chen et al., 2020).

**FIGURE 1:**
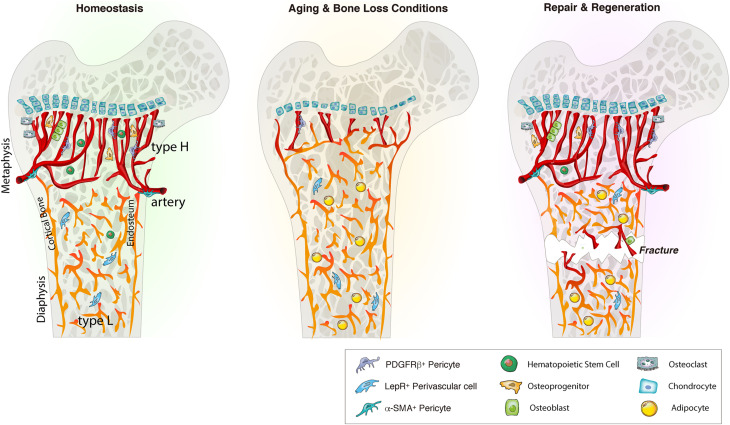
Bone marrow blood vessel organization and niche microenvironment in homeostasis, aging and regeneration. In homeostasis, young bone exhibits an abundance of type H vessels in metaphyseal regions. Type H endothelium is closely associated with osteoprogenitors and stimulates angiogenesis and osteogenesis via angiocrine factors. During aging and bone loss conditions, type H vessel density declines. This decline is accompanied by a reduction osteoprogenitors, reducing osteogenesis and bone mass. Aging also reduces the pool of HSCs while increasing the adipocyte compartment. Bone injury such as fracture or irradiation stimulates type H vessels, osteoprogenitors and HSC proliferation and differentiation to enhance angiogenesis and osteogenesis that guide bone repair and regeneration. LepR, leptin receptor.

While sharing cell surface markers such as E-selectin (Winkler et al., 2012), CD31 (Pecam-1), Endomucin (Kusumbe et al., 2014), VE-Cadherin (Nolan et al., 2007), and Laminin (Nombela-Arrieta et al., 2013), BM ECs can be divided into multiple subpopulations based on the distinct expression pattern of these (Kopp et al., 2009; Castro et al., 2018). Fenestrated sinusoidal capillaries represent the majority of capillaries in the bone. They express low levels of Endomucin and CD31, therefore termed as type L vessels (Kusumbe et al., 2014). Sinusoidal ECs also express vascular endothelial growth factor receptor 3 (VEGFR3). In contrast, arteriolar ECs are negative for VEGFR3 (Kopp et al., 2009; Ramasamy, 2017). Arterial and sinusoidal ECs can be further distinguished with a combination of Podoplanin and Sca-1. Arterial ECs are negative for Podoplanin and express high levels of Sca-1 while sinusoidal ECs are positive for Podoplanin and express low level of Sca-1 (Xu C. et al., 2018). Arterial ECs have been found to be the major source of SCF in the BM and arterial SCF is crucial for HSC function (Xu C. et al., 2018). Type H endothelium, a recently discovered vessel subtype in bone, shows high levels of Endomucin and CD31 (Kusumbe et al., 2014). Type H vessels are mainly located in metaphyseal regions close to the growth plate (Figure 1). They are organized in a columnar fashion and are physically associated with osteoprogenitors (Kusumbe et al., 2014). Due to their direct connection to arterioles, type H vessels contain higher oxygen levels and blood flow than sinusoidal type L vessels (Kusumbe et al., 2014; Ramasamy et al., 2014). Type H vessels are also less permeable than type L vessels, creating an environment with lower ROS levels (Filipowska et al., 2017; Ramasamy, 2017).

These morphological and functional differences between type H vessels, type L/sinusoids and arteries create functionally distinct BM vascular niches, that regulate osteoprogenitor and blood cell proliferation and differentiation, partly via gradients of oxygen tension (Marenzana and Arnett, 2013). The arterial niche contains the surrounding type H vessels in the marrow space, that are identified by the high expression of CD31 and Endomucin (Duarte et al., 2018). Type H vessels also span the endosteum, forming the endosteal vessels (Kusumbe et al., 2014).

Endothelial cell function and stability are closely linked to perivascular cells (Rafii et al., 2016). Different vessel types are supported by distinct perivascular cell types of mesenchymal origin that contribute to these specialized vascular niches (Kusumbe et al., 2016; Ramasamy et al., 2016). For instance, arteries are wrapped with smooth muscle actin (α-SMA). Arterioles and type H capillaries are associated with NG2 and platelet-derived growth factor receptors β (PDGFR-β) expressing pericytes and Nestin-GFP^*bright*^ mesenchymal stem and progenitor cells (MSPCs) (Kunisaki et al., 2013; Mizoguchi et al., 2014). Type H vessels are surrounded by Osterix and Runx2 expressing osteoprogenitors (Figure 1; Kusumbe et al., 2014; Filipowska et al., 2017; Ramasamy, 2017; Peng et al., 2020). Sinusoidal type L vessels are mainly supported by perivascular LepR-expressing cells, that contribute to the adipocyte lineage, and CXCL12-abundant reticular (CAR) cells that support HSCs (Sugiyama et al., 2006; Ding et al., 2012; Boulais and Frenette, 2015).

**FIGURE 2:**
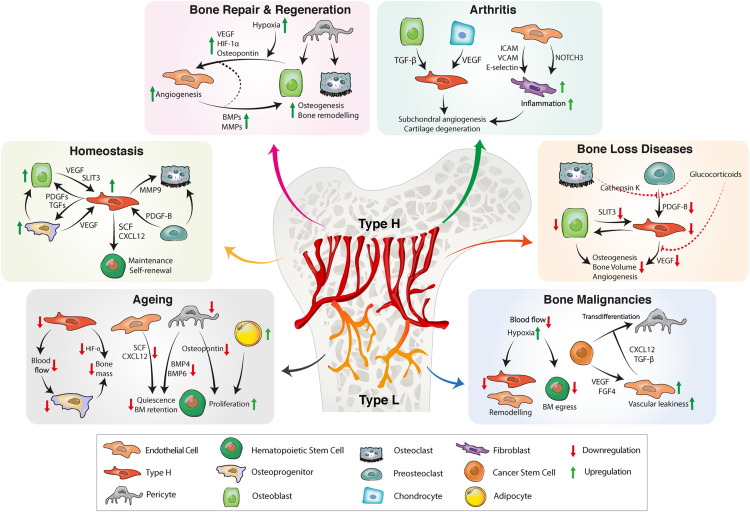
BM vascular niche remodeling in homeostasis, aging, inflammation, and bone diseases. In homeostasis, type H endothelium secretes angiocrine factors to promote osteogenesis, bone remodeling and HSC maintenance. A reduction of type H ECs and pericytes during aging decreases osteogenesis and impairs HSC function. Reduced secretion of proangiogenic factors further results in bone loss. Bone repair requires proangiogenic factors for revascularization and bone formation. CSCs also have the ability to secrete proangiogenic factors that stimulate tumor angiogenesis. Tumor ECs produce proinflammatory cytokines, facilitating vascular niche integration of cancer cells. In inflammatory arthritis, inflamed synovium increases the production of proinflammatory cytokines that trigger inflammation, pathological angiogenesis and cartilage degradation. BM, bone marrow; HSC, hematopoietic stem cell, EC, endothelial cell; CSC, cancer stem cell; FGF, fibroblast-derived growth factor; TGF, transforming growth factor; CXCL12, C-X-C motif chemokine 12; VEGF, vascular endothelial growth factor; SLIT3, slit guidance ligand 3; BMP, bone morphogenetic protein; PDGF, platelet-derived growth factor; SCF, stem cell factor; ICAM, intercellular adhesion molecule; VCAM, vascular cell adhesion protein; MMP, matrix metalloproteinase; HIF, hypoxia-inducible factor.

Hematopoietic stem cells preferentially localize within the vascular niches throughout the BM. However, the exact location of HSCs within the distinct vascular niches is still unsettled with new studies debating of HSC localization. Imaging studies of HSCs in the BM have produced different results. Using the HSC markers α-catulin and c-kit for deep confocal imaging of the BM, showed that the majority of dividing and non-dividing HSCs are localized in the central diaphyseal BM around sinusoidal blood vessels and are distant from bone surfaces and arteriolar vessels (Acar et al., 2015). Analysis of distinct subsets of HSCs demonstrated a preferential location of quiescent HSCs near endosteal arteriolar vessels surrounded by NG2^+^ pericytes. In contrast, proliferative HSCs move away from arterioles toward LepR^+^ perisinusoidal niches, suggesting a pivotal role for arteriolar niches in maintaining HSC quiescence and a distinct HSC distribution between differential BM niches (Kunisaki et al., 2013; Itkin et al., 2016; Figure 2). Recent intravital imaging studies of genetically labeled native HSCs suggest that LT-HSCs reside near sinusoidal vessels in the endosteum and exhibit limited motility (Christodoulou et al., 2020). In contrast, another recent study found that the majority of HSCs are localized in the perivascular space with significant motility and spatial association with SCF-expressing stromal cells (Upadhaya et al., 2020). The above studies were based on different mouse models, or different cell surface markers were used, which may ultimately lead to the analysis of different subsets of HSCs. Overall, the detailed location of HSCs in their vascular niches requires further investigation.

## Vascular Sensing and Signaling in the Bone Marrow Microenvironment

Bone marrow ECs and perivascular stromal cells express a range of paracrine factors and interact with surrounding cells to maintain vascular tissue homeostasis and create vascular stem cell niches. These factors include cytokines and growth factors and are collectively termed angiocrine factors (Table 1). Some of them are produced constitutively, while other factors modulate the production of angiocrine factors (Rafii et al., 2016). Angiocrine signals enable crosstalk between ECs and neighboring cell types, thereby contributing to various tissue functions, including maintenance of tissue homeostasis and regulation of stem cell behavior and differentiation (Ding et al., 2012; Sivan et al., 2019; Chen et al., 2020). BM ECs promote HSC maintenance and self-renewal and blood vessel formation by expressing stimulating factors such as CXCL12, SCF, and vascular endothelial growth factor (VEGF) (Sugiyama et al., 2006; Coskun and Hirschi, 2010). Expression of cytokines, such as granulocyte colony-stimulating factor (G-CSF) and various interleukins, enables BM ECs to initiate lineage-specific HSPC differentiation (Kobayashi et al., 2010; Rafii et al., 2016). Furthermore, angiocrine signaling plays an essential role in the coupling of bone angiogenesis and osteogenesis (Chen et al., 2020). This osteo-angiogenic coupling is mediated explicitly by type H ECs that release various osteogenesis stimulating factors such as platelet-derived growth factor B (PDGF-B) and VEGF (Maes and Clemens, 2014; Grosso et al., 2017; Rumney et al., 2019).

**TABLE 1:** Vascular niche associated factors in bone aging, stress, and disease.

Sl. No	Factor/Signal	Function	Cell Type	Condition	References
1	Angiopoietins	Inhibition of angiogenesis	Endothelial cells	Osteosarcoma	Habel et al., 2015
2	BMP-4 BMP-6	Cancer cell dormancy	CSCs, HSCs	Bone metastasis, Ageing	Singh et al., 2019
3	Cathepsin K	Inhibition of PDGF-BB secretion	Pre-osteoclasts	Osteoporosis	Yang et al., 2018
4	CXCL12	HSC maintenance, Chemoresistance, tumor proliferation	HSC, Endothelial Cell	Ageing, Bone malignancies	Kusumbe et al., 2016; Poulos et al., 2017
5	CYR61	Primary tumor vascularization, VEGFA production	Endothelial cell, CSCs	Osteosarcoma	Habel et al., 2015
6	Dll4	HSC differentiation and maintenance	HSCs	Irradiation, chemotherapy	Tikhonova et al., 2019
7	FGF4	Endothelial activation	Endothelial cells	Bone malignancies	Cao et al., 2014
8	G-CSF GM-CSF	Angiogenesis, HSC differentiation	HSCs, endothelial cells	Irradiation, chemotherapy	Kobayashi et al., 2010
9	HIF-1α	Angiogenesis, osteogenesis	Endothelial cells	Ageing, Bone repair	Kusumbe et al., 2014
10	IL-1 TNF-a	HSC differentiation & migration, inflammation	HSCs, Endothelial cells	Ageing, Inflammation	Broudy et al., 1986; Sieff et al., 1987; Boettcher et al., 2014
11	IL-6	Downregulation of inflammatory response	Endothelial cells	Inflammation	Luu et al., 2013
12	Jagged1	Angiogenesis, HSC regeneration	Endothelial cells, HSCs	Irradiation, chemotherapy	Kobayashi et al., 2010
13	MMPs	Cartilage matrix remodeling, tumor invasion	Chondrocytes	Osteoarthritis, bone repair, Bone metastasis	Behonick et al., 2007; Wang X. et al., 2013; Romeo et al., 2019
14	mTORC1	Subchondral angiogenesis, VEGFA production	Endothelial cells	Osteoarthritis	Lu et al., 2018
15	Notch	Angiogenesis Vascular niche function	Endothelial cells, HSCs	Ageing	Kusumbe et al., 2016; Poulos et al., 2017
16	NOTCH3	Differentiation & expansion of synovial fibroblasts	Synovial fibroblasts	Rheumatoid arthritis	Wei et al., 2020
17	Osteopontin	Angiogenesis	Endothelial cells, pericytes	Bone repair	Duvall et al., 2007
18	PDGF-BB	Susceptibility to radiation and chemotherapy Osteo-angiogenic coupling	Pericytes	Bone metastasis, Ageing, Osteoporosis	Xie et al., 2014; Yang et al., 2018; Singh et al., 2019
19	PECAM	Inhibition of angiogenesis	Endothelial cells	Osteosarcoma	Habel et al., 2015
20	PEDF	Inhibition of tumor angiogenesis and growth	Endothelial cells	Osteosarcoma	Cai et al., 2006; Ek et al., 2007b
21	SCF	HSC maintenance	HSC	Ageing	Kusumbe et al., 2016; Poulos et al., 2017
22	SLIT3	Angiogenesis	Endothelial cells	Osteoporosis	Xu R. et al., 2018
23	TGF-β1	Type H formation	Endothelial cells	Osteoarthritis	Brenet et al., 2013; Zhen et al., 2013
24	Thrombospondin-1	Cancer cell dormancy, Inhibition of angiogenesis	Endothelial cells, DTCs	Bone metastasis, Osteoporosis	Rae et al., 2009; Ghajar et al., 2013
25	VCAM1 ICAM E-selectin	Immune cell recruitment	Fibroblasts	Rheumatoid arthritis	Klimiuk et al., 2002
26	VEGFA	(Tumor) angiogenesis Osteogenesis	Endothelial cell	Ageing, Bone malignancies, inflammation	Kusumbe et al., 2016; Passaro et al., 2017; Duarte et al., 2018
27	VEGFR1	Pre-metastatic niche	CSCs	Bone metastasis	Kaplan et al., 2005
28	VEGFR2	Osteogenesis, chondrogenesis, sinusoidal regeneration	Osteoblasts, sinusoidal endothelial cells	Bone repair, irradiation	Tarkka et al., 2003; Hooper et al., 2009

**FIGURE 3:**
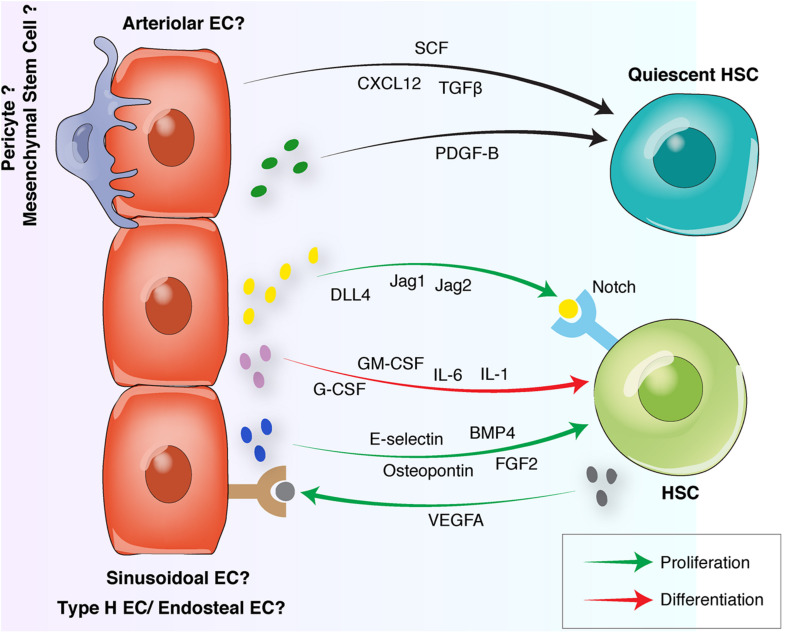
Endothelial interactions with HSCs in the BM vascular niche. Quiescent HSCs preferentially locate in arteriolar niches surrounded by NG2^+^ pericytes. Arteriolar ECs and NG2^+^ pericytes release quiescence-inducing factors including SCF, CXCL12, and PDGF-B. Proliferative HSCs move away from arterioles toward sinusoidal niches. Sinusoidal ECs to release proliferative factors such as Osteopontin, FGF-2, E-selectin, and Notch ligands. HSC differentiation is induced by the endothelial release of G-CSF, GM-CSF or interleukins. Reciprocally, HSCs can induce endothelial proliferation by releasing proangiogenic factors such as VEGFA. HSC, hematopoietic stem cell, EC, endothelial cell; FGF, fibroblast-derived growth factor; TGF, transforming growth factor; CXCL12, C-X-C motif chemokine 12; VEGF, vascular endothelial growth factor; BMP, bone morphogenetic protein; PDGF, platelet-derived growth factor; SCF, stem cell factor; G-CSF, granulocyte colony-stimulating factor; GM-CSF, granulocyte-macrophage colony-stimulating factor.

Vascular endothelial growth factor A (VEGFA) is one of the most important proangiogenic factors in physiological and pathological conditions. It is secreted by various cells including ECs and bone lineage cells to promote EC migration and proliferation and couple bone angiogenesis to bone remodeling (Tombran-Tink and Barnstable, 2004; Sivan et al., 2019; Figure 3). VEGFA has also been shown to be produced by HSCs (Gerber and Ferrara, 2003). VEGFA is released in a paracrine and autocrine fashion and primarily binds to VEGFR2, which functions as a critical regulator of endothelial proliferation and migration (Podar and Anderson, 2005). EC-specific deletion of this receptor impairs angiogenesis, reduces vessel density and disrupts metaphyseal organization (Wang L. et al., 2013). Further, loss of VEGFA can disrupt vessel invasion into hypertrophic chondrocytes and impair osteogenesis and bone growth (Maes et al., 2002). *In vitro* studies suggest that angiogenic and osteogenic effects of VEGFA rely on tight regulation of timing and dose. For instance, VEGF-mediated activation of VEGFR2 suppresses PDGFR-β signaling via formation of a VEGFR2/PDGFR-β complex, resulting in a loss of pericytes and impaired vessel stability (Greenberg et al., 2008). Independent of its angiogenic function, VEGFA has also been implicated in HSC specification via loss of Notch1 expression (Leung et al., 2013; Figure 2).

PDGFR-β signaling also contributes to osteo-angiogenic coupling. PDGF-B is secreted by ECs and pre-osteoclasts and stimulates mesenchymal and endothelial migration and proliferation via binding to its receptor PDGFR-β (Xie et al., 2014; Xu R. et al., 2018; Peng et al., 2020; Figure 3). By activating the focal adhesion kinase (FAK) pathway, PDGF-B also induces type H vessel formation and osteogenesis (Xie et al., 2014).

Hypoxia-inducible factor (HIF) is a transcription factor that regulates cellular signaling in response to changes in oxygen levels (Riddle et al., 2009; Peng et al., 2020). HIF-1α is one of three α-subunits with hypoxia-dependent activity (Semenza, 1999) and acts as an essential regulator of physiological and pathological bone angiogenesis, osteogenesis, and regeneration (Greijer et al., 2005; Wan et al., 2008). Under hypoxic conditions, ECs and osteoblasts increase HIF-1α expression, which promotes the expression of VEGFA and other proangiogenic factors (Kwon et al., 2011; Peng et al., 2020). In the metaphysis, HIF-1α is expressed by type H vessels in an oxygen-independent manner. Pharmacological and genetic activation of HIF signaling promotes type H vessel formation and osteogenesis. EC inactivation of HIF-1α impairs type H vessel number and bone formation (Kusumbe et al., 2014). In contrast, EC-specific deletion of Von Hippel Lindau (Vhl), which stabilizes endothelial HIF-1α, enhances the abundance of type H vessels and osteoprogenitors (Jaakkola et al., 2001). Similarly, pharmacological stabilization of HIF-1α via administration of deferoxamine mesylate promoted type H vessel formation and increased osteoprogenitors and osteoblasts (Jones and Harris, 2006; Kusumbe et al., 2014). A follow up study demonstrated that the transcriptional coregulators Yap1 and Taz negatively regulate bone angiogenesis by suppressing endothelial HIF-1α activity. EC-specific inactivation of Yap1 and Taz upregulated HIF-1α target genes and increased type H and type L vessel density, which could be normalized with EC-specific stabilization of HIF-1α (Sivaraj et al., 2020).

C-X-C motif chemokine 12 is a crucial chemokine in HSC and lymphoid progenitor maintenance and quiescence. It is expressed by BM ECs, perivascular cells, osteoblasts and sympathetic neuronal cells (Ding and Morrison, 2013; Sivan et al., 2019) and particularly Nestin^+^ perivascular stromal cells that are physically associated with HSCs (Mendez-Ferrer et al., 2010; Figure 2). Selective ablation of CXCL12-expressing perivascular cells significantly decreased HSC numbers and size (Omatsu et al., 2010). EC-specific deletion of CXCL12 also reduces HSC frequency (Ding and Morrison, 2013; Boulais and Frenette, 2015) and impairs long-term HSC repopulation activity. Deletion of CXCL12 in MSPCs has similar effects on HSC number and repopulation activity, indicating a crucial role of endothelial and perivascular derived CXCL12 in supporting HSCs (Greenbaum et al., 2013; Boulais and Frenette, 2015).

Stem cell factor is another crucial niche component for HSC maintenance. Via differential splicing and proteolytic cleavage, it is found in a soluble and a membrane-bound form. Deletion of the expression of membrane-bound SCF significantly depletes HSCs, demonstrating a particularly important role of membrane-bound SCF for HSC maintenance (Barker, 1994; Ding et al., 2012; Figures 2, 3). SCF is expressed by perivascular stromal cells and ECs. Arterial ECs, type H ECs and sinusoidal ECs; all express SCF with higher expression levels detected in the type H and arterial ECs (Ding et al., 2012; Kusumbe et al., 2016; Sivan et al., 2019). Within the mesenchymal compartment; deletion of SCF from Nestin-GFP cells resulted in the depletion of HSCs (Asada et al., 2017) indicating that SCF from these peri-arterial mesenchymal stem cells is critical for HSC maintenance. Further, conditional deletion of SCF in ECs using Tie2-Cre which marks the arterial, type H and sinusoidal ECs and the LepR-Cre mice which marks the perivascular cells, also led to a depletion of BM and spleen HSCs (Ding et al., 2012). These findings suggest an essential role for endothelial and perivascular SCF in HSC survival and maintenance.

The proximity of BM ECs to stem and progenitor cells in different tissues facilitates the delivery of angiocrine factors and cell-to-cell contacts (Rafii et al., 2016). One of the most critical cellular interaction mechanisms is the Notch signaling pathway that controls the cell fate decisions via binding of Notch ligands of the Delta-like or Jagged family to Notch receptors (Milner and Bigas, 1999; Fernandez et al., 2008). Opposite to its function in other organs and tissues, EC Notch activation in long bones stimulates EC proliferation and type H vessel formation (Ramasamy et al., 2014). Endothelial Notch activation also enhances HSCs and PDGFR-β and NG2 expressing perivascular cells and increases SCF levels, indicating a Notch-mediated enhancement of vascular niche function (Figure 2; Kusumbe et al., 2016; Sivan et al., 2019). Most of the research into vascular niches and HSC maintenance has been conducted using mouse models. In contrast, identifying factors that maintain human HSCs has been more challenging (Sugimura, 2018). A study has identified the compounds UM171 and SR1 from a family of chemically related small molecules to stimulate human HSC expansion *in vitro* (Fares et al., 2014). However, the precise downstream mechanisms of these compounds remain unclear. Using a vascular niche reconstitution approach; human endothelial and mesenchymal progenitors and HSCs were implanted in mice to create a humanized BM xenotransplantation model that may help to identify factors for the maintenance of human HSCs (Reinisch et al., 2016).

## Aging of the Bone Marrow Vascular Niche

During aging, the BM endothelium exhibits significant morphological and metabolic changes. Imaging and flow cytometry studies report an overall decrease in the arteriolar proportion (Kusumbe et al., 2016; Maryanovich et al., 2018; Ho et al., 2019). This significant reduction of arteriolar vessels and PDGFR-β expressing perivascular stromal cells is concomitant with a decrease in SCF levels in long bones of aged mice (Kusumbe et al., 2014; Figure 1 and Table 1). In contrast, the abundance of sinusoidal type L blood vessels remain unchanged during aging (Kusumbe et al., 2014; Ho et al., 2019).

Along with a decline in type H vessel density, aged bones show a decrease in blood flow that is likely to induce metabolic changes in the BM microenvironment (Figure 3; Ramasamy et al., 2016; Sivan et al., 2019). Age-related type H vessel decline correlates with a reduction of osteoprogenitors, osteogenesis and bone density (Kusumbe et al., 2014) and endosteal BM niches (Ho et al., 2019; Figures 1, 3). Further metabolic changes in aged BM endothelium include increased hypoxia and reactive oxygen species (ROS) levels, which are associated with decreased angiogenic and migration potentials (Poulos et al., 2017). Aging also reduces the endothelial expression of HIF-1α, contributing to the age-related loss of bone mass and type H endothelium (Kusumbe et al., 2014; Figure 3 and Table 1). BM ECs of aged mice express significantly lower levels of pro-hematopoietic factors in comparison to BM ECs of young mice. These factors include CXCL12, SCF and Notch ligands that are critical for HSC homeostasis (Kusumbe et al., 2016; Poulos et al., 2017; Table 1). Moreover, Notch activity is higher in type H ECs and adjacent perivascular cells, suggesting a link between the age-related decrease of endosteal vessels and impairments in the endothelial Notch signaling pathway along with the loss of type H vessels with age. Consistent with this, activation of endothelial Notch signaling enhances blood flow to the bones and increases HSC abundance (Kusumbe et al., 2016).

In addition to functional and metabolic changes, aged endothelium shows significant morphological changes, including augmented vasodilation and leakiness and impaired vascular integrity (Poulos et al., 2017).

Perivascular cells also exhibit functional changes during aging. Aging reduces the abundance of pericytes, thereby decreasing the release of quiescence inducing factors such as SCF, Bmp4, and Bmp6, which ultimately results in the loss of HSC and cancer cell quiescence (Singh et al., 2019; Figure 3 and Table 1). Further, reduced perivascular secretion of Osteopontin may induce HSC proliferation and increase HSC numbers during aging (Guidi et al., 2017; Figure 3 and Table 1). BM MSCs show reduced proliferation and a bias toward adipogenesis at the expense of osteogenic differentiation (Kim et al., 2012; Singh et al., 2016). Since adipocytes are known to inhibit HSC function and B-lymphomagenesis (Kennedy and Knight, 2015), age-related adipocyte accumulation in the BM may underlie myeloid skewing and impaired functionality of aged HSCs (Kovtonyuk et al., 2016).

Despite the increase in HSC numbers, HSC function has been found to decrease during aging, showing a reduction of self-renewal and loss of quiescence (Morrison et al., 1996; Chambers et al., 2007; Pang et al., 2011; Kovtonyuk et al., 2016; Singh et al., 2019). These functional HSC alterations correlate with an age-related relocation of HSCs away from endosteal arteriolar niches, favoring non-endosteal sinusoidal BM niches in the central BM (Maryanovich et al., 2018; Ho et al., 2019; Saçma et al., 2019). Cell-intrinsic dysregulations such as protein misfolding and accumulation of DNA damage have been thought to underlie these age-related HSC changes (Beerman et al., 2014; Flach et al., 2014; Kovtonyuk et al., 2016). However, there is increasing evidence that changes in the BM microenvironment contribute to reduced HSC function during aging (Kovtonyuk et al., 2016). Aging of the BM vascular niche disrupts HSC homeostasis and is sufficient to induce an aging-associated HSC phenotype *in vitro* and *in vivo* (Poulos et al., 2017). Co-cultures of young HSCs with aged ECs and *in vivo* infusion of aged ECs after myelosuppression impair hematopoietic repopulating activity and induce a myeloid lineage bias. Infusion of young ECs improves repopulation activity and partially restores the HSC function, suggesting a reciprocal relationship between HSC aging and vascular niche alterations (Poulos et al., 2017). Hematopoietic aging leads to a decreased functionality of the immune system. Immunological impairments lead to increased susceptibility to infections, autoimmune disorders, and hematological malignancies (Kim et al., 2003; Esplin et al., 2011; Kovtonyuk et al., 2016) that cause additional vascular niche alterations and further disrupt HSC homeostasis.

## Inflammation Driven Modulation of the Bone Vasculature

Inflammation is a protective response of the immune system against different inflammatory stimuli such as tissue injury, physical stress or infection. Upon peripheral infection, pattern recognition receptors (PRRs) on ECs, MSCs and other hematopoietic and non-hematopoietic cells are activated. ECs express various PRRs, including toll-like receptors (TLRs), that enable them to detect systemic infections and regulate inflammatory responses. Upon TLR4 activation or stimulation with IL-1 or TNF-α, ECs upregulate their production of proinflammatory cytokines such as interleukins, TNF-α, G-CSF, and granulocyte-macrophage colony-stimulating factor (GM-CSF) (Broudy et al., 1986; Sieff et al., 1987; Boettcher et al., 2014) that circulate to the BM. In the BM, they induce HSC proliferation, migration, and differentiation to restock the pool of immune effector cells of the inflammatory response (Kovtonyuk et al., 2016). G-CSF and GM-CSF also promote granulopoiesis, and proliferation and recruitment of neutrophils (Schuettpelz et al., 2014; Kovtonyuk et al., 2016). Proinflammatory cytokines also stimulate ECs and MSCs to increase cytokine production and secretion, creating a positive feedback loop (Luu et al., 2013; Kovtonyuk et al., 2016). Activated ECs increase their expression of adhesion molecules such as vascular cell adhesion molecule 1 (VCAM1), further facilitating the immune cell recruitment (Castro et al., 2018). Upregulated expression of the Notch ligand Jagged-2 by the bone endothelium in response to the proinflammatory stimuli LPS and TNF-α, suggests that BM ECs may promote hematopoietic progenitor cell proliferation via increased Notch activation (Fernandez et al., 2008).

Inflammation distinctively alters both morphology and function of the BM endothelium. During acute inflammation, IFN-α promotes activation and proliferation of BM ECs is mediated by increased VEGF production by hematopoietic cells such as HSCs (Prendergast et al., 2017; Batsivari et al., 2020). In line with increased angiogenic VEGF levels, enhanced number of sinusoids and luminary are found in the inflamed BM. Inflammation also increases hypoxic regions in long bones, which may contribute to enhanced bone angiogenesis (Vandoorne et al., 2018). Furthermore, the BM endothelium shows high vascular permeability and leakiness during inflammation, caused by the opening of tight junctions in order to promote *trans*-endothelial migration of immune cells (Prendergast et al., 2017; Vandoorne et al., 2018; Batsivari et al., 2020).

Inflammation-driven niche alterations show many similarities to changes in the aged BM niche. Elevated levels of proinflammatory cytokines have been associated with aging of the BM microenvironment and age-related myeloid malignancies. Both inflammation and aging induce a myeloid differentiation bias and impair HSC self-renewal capacity (Kovtonyuk et al., 2016). Serum levels of proinflammatory cytokines such as IL-1, IL-6, and TNF-α are upregulated in the aged population, and this upregulation may underlie the high myelopoiesis and adipogenesis that occurs in aged BM (Hasegawa et al., 2000; Ferrucci et al., 2005; Table 1). Age-associated chronic inflammatory cytokine production, termed as “inflammaging,” is proposed to result in cumulative tissue damage (Franceschi et al., 2000; Baylis et al., 2013). Myeloid cells and adipocytes represent significant sources of proinflammatory cytokines, suggesting a positive feedback loop between aging and inflammation. During aging, increased levels of pro-inflammatory cytokines create a chronic proinflammatory state that further enhances myeloid skewing of HSCs (Ergen et al., 2012). In addition, adipogenic differentiation of perivascular MSCs may also promote myelopoiesis and cytokine production that is observed during aging (Kovtonyuk et al., 2016). To uncover the contributions of vascular and perivascular cells during age-associated inflammation, the inflammaging process needs further investigation.

## Vascular Perturbations in Arthritis

Alterations in vascularization in connective tissues including bone have been described in a number of arthropathies; neoangiogenesis has been proposed as a pathological process in some. Here, rheumatoid arthritis (RA) as an exemplar of chronic inflammatory arthritis and osteoarthritis as the commonest degenerative joint disorder are given.

### Rheumatoid Arthritis

Rheumatoid arthritis is the most common form of chronic inflammatory arthritis and results in joint inflammation, articular bone loss and increased cardiovascular morbidity and mortality (Totoson et al., 2016). RA is characterized by increased articular angiogenesis and synovial inflammation that damages affected joints (Walsh et al., 2010). Proinflammatory cytokines from inflamed joints activate ECs by inducing endothelial expression of VCAM1, intracellular adhesion molecule 1 (ICAM1), E-selectin and other adhesion molecules (Klimiuk et al., 2002; Kong et al., 2018; Figure 3 and Table 1). Cell adhesion molecules facilitate leukocyte and fibroblast invasion into the joint and shift ECs into a proinflammatory state (Klimiuk et al., 2002; Bordy et al., 2018; Sivan et al., 2019). Endothelial activation and systemic inflammation trigger endothelial dysfunction, which is characterized by impaired vasodilation. Endothelial dysfunction crucially contributes to the development of accelerated atherogenesis and cardiovascular mortality in RA (Ulker et al., 2000; Totoson et al., 2014). Impaired vasodilation increases the blood flow and pressure that is transmitted into microvessels, thereby damaging vascular beds (Totoson et al., 2016; Bordy et al., 2018). Multiple studies have identified a decreased bioavailability of vasoactive nitric oxide (NO) underlying impaired vasodilation in RA (Wilcox et al., 1997; Garg et al., 2017). During the early stage of RA, this is compensated by increased NO synthase activity that is lost with persistence of inflammation and the onset of endothelial dysfunction (Totoson et al., 2016).

Transcriptomic analysis of human synovial fibroblasts has identified a distinct subpopulation characterized by the expression of Podoplanin, THY1, and Cadherin-11 (Mizoguchi et al., 2018). This fibroblast subpopulation is significantly upregulated in RA patients, localizing and expanding near the blood vessels and secreting proinflammatory cytokines (Mizoguchi et al., 2018). Upregulation of NOTCH3 and Notch target genes via blood vessels is found in active RA (Wei et al., 2020; Figure 3 and Table 1). Deletion of Notch3 or blocking of NOTCH3 signaling blocks fibroblast expansion, alleviates inflammation and prevents damage of inflamed joints, indicating a critical role for Notch3 signaling in regulating synovial fibroblast differentiation, expansion and disease activity (Wei et al., 2020). Moreover, Notch-1 has been shown to mediate VEGF/Angiopoietin 2-induced angiogenesis and EC invasion in RA synovial explants (Gao et al., 2013).

Levels of neoangiogenesis in RA appear to be closely linked to levels of synovial inflammation and to pain experienced, making it a viable therapeutic target in this disease (Paleolog, 2009; Fransès et al., 2010; Ashraf et al., 2011). Proinflammatory cytokines also modulate osteoclastogenesis and impair osteoblastic bone repair, facilitating articular bone loss in RA if inflammation is not controlled pharmacologically (Karmakar et al., 2010). At a local level this can lead to bone erosion, loss and deformity, and systemically to osteopenia and osteoporosis. Controlling inflammation by inhibiting TNF-α significantly improves flow-mediated vasodilation and disease activity and may reduce cardiovascular morbidity (Jacobsson et al., 2005; Greenberg et al., 2011; Kawalkowska et al., 2019; Végh et al., 2020).

### Osteoarthritis

Osteoarthritis (OA) is a chronic joint disease and considered the most common form of arthritis (Ashford and Williard, 2014; Vincent and Watt, 2018; Hunter and Bierma-Zeinstra, 2019). OA is characterized by articular cartilage loss and new bone formation (osteophytosis) associated with sclerosis of underlying bone. BM abnormalities are seen including BM edema (BME), abnormal osteogenesis and a reported increase in subchondral angiogenesis (Felson et al., 2003; Li et al., 2013). Some believe that these subchondral bone changes contribute to the progressive degeneration of cartilage, although this is not well understood (Dyke et al., 2015). Disruption of subchondral blood flow impairs diffusion of nutrients to the articular cartilage, resulting in the death of osteocytes and joint damage (Zhen et al., 2013; Glyn-Jones et al., 2015; Sivan et al., 2019). OA has also been associated with increased cardiovascular comorbidity and mortality (Turkiewicz et al., 2019). One study of patients with knee OA revealed an association between OA radiological severity and increased arterial stiffness (Tootsi et al., 2016).

Obesity is known to increase the incidence and progression of OA (King et al., 2013). Multiple studies demonstrate increased adipokines including serum leptin levels associated with the disease (de Boer et al., 2012; Tootsi et al., 2016; Morales Abaunza et al., 2020). Leptin has been shown to upregulate proteolytic enzymes such as MMP-1 and MMP-3 in articular cartilage and correlates with their levels in the synovial fluid of OA patients, suggesting a potential for an enhanced catabolic effect on OA cartilage (Koskinen et al., 2011).

Inflammatory cytokines such as TNF-α, IL-6, IL-11, and IFN-γ have been reported to be upregulated in the synovial fluid, the articular cartilage and synovium of OA patients (Zhou et al., 2016). Inflammatory signaling in connective tissues (which can be driven by such inflammatory cytokines as well as by mechanical stress to tissues) is associated with protease expression such as aggrecanases and metalloproteinases (MMPs) and chemokines, driving cartilage degeneration (Findlay and Kuliwaba, 2016). Increased activation of TGF-β1 in murine and human OA effectively recruits MSCs and type H vessels, causing OA-characteristic abnormal bone formation and augmented subchondral angiogenesis (Zhen et al., 2013; Figure 3 and Table 1). Osteoblast-specific overexpression of TGF-β1 induces murine OA (Blaney Davidson et al., 2007; Zhen et al., 2013), while subchondral inhibition of TGF-β and type H vessel formation alleviates cartilage degeneration (Cui et al., 2016).

Increased subchondral angiogenesis may contribute to OA progression (Hamilton et al., 2016). Type H vessels can drive OA progression by releasing proteases such as MMP-2, MMP-9, and MMP-14, which promote the resorption of cartilage matrix and its degeneration (Romeo et al., 2019; Table 1). Articular chondrocytes *in vitro* stimulate excessive subchondral type H vessel formation by mechanistic target of rapamycin complex 1 (mTORC1)-mediated VEGFA production (Lu et al., 2018). Reciprocally, vascular-derived nutrients promote chondrocyte and mTORC1 activation and VEGF production, further enhancing subchondral angiogenesis (Lu et al., 2018; Figure 3). Inhibition of mTORC1 is able to reduce this pathological angiogenesis, thereby delaying disease progression (Lu et al., 2018). During later stages of OA, articular cartilage, synovium and subchondral bone show increased levels of VEGF (Hamilton et al., 2016). Synovial VEGF levels have also been found to correlate with disease severity and pain in patients with knee OA, potentially implicating VEGF as a biomarker for OA pathogenesis (Gaballah et al., 2016). Sensory nerves grow along new blood vessels in osteoarthritic joints, eventually reaching non-calcified articular cartilage, osteophytes and menisci, and may be a source of pain from all of these structures. Angiogenesis could therefore be a source of pain in OA (Mapp and Walsh, 2012).

## Angiogenesis and Bone Pain

Inflamed synovium has upregulated levels of the neurotrophin nerve growth factor (NGF) (Aloe et al., 1999). NGF signals by way of binding its two receptors TrkA and p75. Most pain signaling is considered to be via TrkA whilst p75 signaling relates to cell survival/death (Reichardt, 2006). NGF signaling is known to be increased in the context of inflammation, for example in RA (Skaper, 2017). In OA, NGF stimulates sensory nerve growth into vascular channels of articular cartilage and subchondral bone, contributing to arthritic pain (Suri et al., 2007). Neutralizing NGF with monoclonal antibodies in both murine and human OA leads to reduction in joint pain (Lane et al., 2010; McNamee et al., 2010). Substantial expression of NGF receptors TrkA and p75 is found in rat bone (Nencini et al., 2017). NGF injection into rat bone rapidly activates nociceptors and produces an acute behavioral pain response, implicating NGF in inflammatory bone pain (Nencini et al., 2017; Table 1). NGF also functions as an angiogenic factor. ECs express TrkA and p75 and administration of NGF induces capillary sprouting and increased neuronal VEGF expression in newborn rats (Calzà et al., 2001). NGF receptor binding activates the mRas/ERK and PI3K/Ak pathways that control endothelial proliferation and survival. VEGF activates these same pathways, perhaps suggesting a joint role for NGF and VEGF in the regulation of angiogenesis (Nico et al., 2008).

## Vascular Function in Bone Loss Diseases

Osteoporosis is a metabolic bone disease that results in progressive bone loss and fragility (Cooper et al., 2006; Sözen et al., 2017). It is characterized by the imbalance of osteoblast-mediated bone formation and osteoclast-mediated bone resorption with resultant reduction in bone mass, disrupted microarchitectural integrity and increased risk of fracture (Sözen et al., 2017; Compston et al., 2019). Postmenopausal women have an increased susceptibility to osteoporosis, in part due to the fall in estrogen levels at the time of menopause that leads to a higher rate of bone resorption than formation (Ji and Yu, 2015). Aging in both men and women is also associated with increased risk of osteoporosis. Multiple studies have shown a reduction in bone vasculature and bone-forming cells in mouse models of osteoporosis (Weinstein et al., 2010; Cui et al., 2012; Yang et al., 2018). Specifically, age-related loss of type H endothelium appears to play an important role in the pathogenesis of osteoporosis (Ding et al., 2020; Figures 1, 3). Significant reduction of type H vessels is observed in ovariectomized female mice, a commonly used experimental model of postmenopausal osteoporosis (Wang et al., 2017). Likewise, the decline of human type H endothelium is observed in women after menopause (Zhu et al., 2019). A reduction of type H vessels, mature osteoblasts and osteocytes is also observed in a mouse model of glucocorticoid-induced osteoporosis (GIO) (Yang et al., 2018). Glucocorticoids decrease blood flow and inhibit angiogenesis by reducing VEGF levels (Wang et al., 2010; Weinstein et al., 2010) and increasing thrombospondin-1 (Rae et al., 2009; Yang et al., 2018).

Pre-osteoclast PDGF-B secretion induces type H vessel growth and angiogenesis and osteogenesis (Dou et al., 2018; Ding et al., 2020). The osteoclast-derived cathepsin K decreases pre-osteoclast secretion of PDGF-B; this impairs the recruitment of mesenchymal and endothelial progenitors to bone remodeling sites and reduces bone and blood vessel formation (Yang et al., 2018; Figure 3 and Table 1). Interestingly, knockout of cathepsin K in GIO mice prevents PDGF-B reduction and loss of osteoblasts, osteoclasts, and type H ECs. In line with these findings, inhibition of cathepsin K via administration of an inhibitor L-235 prevents osteoporosis and maintains osteoblasts and bone volume (Yang et al., 2018). Moreover, cathepsin K inhibition increases PDGF-B and preserves type H vessel by enhancement of endothelial VEGF production (Yang et al., 2018). Another study implicated a role for another osteoblast-derived proangiogenic factor slit homolog 3 protein (SLIT3) in osteoporosis-associated loss of bone mass and vasculature (Figure 3 and Table 1). Intravenous SLIT3 injection in ovariectomized mice reverses bone loss and augments type H ECs (Xu R. et al., 2018; Ding et al., 2020).

## Dynamics of Bone Vasculature During Fracture Repair

Bone fracture disrupts the typical bone architecture, vasculature, and surrounding tissue. Fractures are often accompanied by blood vessel damage, thereby causing hemorrhage, local hypoxia and susceptibility to infection (Marenzana and Arnett, 2013; Baker et al., 2018). The initial proinflammatory state stimulates cell proliferation and differentiation via expression of IL-1 (Lange et al., 2010), MMP-9 (Wang X. et al., 2013), and BMPs (Cheng et al., 2003; Bahney et al., 2015). A soft callus is formed soon after fracture which stabilizes the site of injury (Baker et al., 2018).

Local hypoxia and high lactate levels after fracture upregulate the expression of HIF1-α and its downstream target VEGF that stimulate angiogenesis and osteogenesis and replace soft callus by vascularized hard callus (Wang et al., 2007; Marenzana and Arnett, 2013; Baker et al., 2018; Bahney et al., 2019; Figure 3 and Table 1). Disruption of osteoblast-derived HIF1-α delays callus formation and impairs fracture healing (Wang et al., 2007).

Angiogenesis is considered to be essential in fracture repair (Hausman et al., 2001). During the repair phase, VEGF stimulates the regrowth of blood vessels into the site of injury to restore normal oxygen and nutrient supply and activate osteoblast function (Marenzana and Arnett, 2013; Figure 1). While inhibition of VEGFR1 and VEGFR2 impairs osteogenesis and chondrogenesis and reduces callus formation (Jacobsen et al., 2008), VEGF administration significantly accelerates fracture repair (Tarkka et al., 2003; Table 1). TNF-α administration has also been shown to promote fracture repair by recruiting muscle-derived stromal cells and promoting osteogenic differentiation (Glass et al., 2011). Regrowth of sensory nerve fibers is stimulated by NGF, creating pain sensation. NGF also stimulates VEGFA-mediated revascularization and promotes ossification via TrkA-mediated communication between sensory nerves and osteoblasts (Tomlinson et al., 2017; Li et al., 2019; Table 1). Inhibition of TrkA signaling reduces nerve regrowth and revascularization, delaying ossification of fracture calluses (Li et al., 2019).

With revascularization of the injury site, new bone tissue is formed directly via progenitor differentiation into osteoblasts (intramembranous ossification) and indirectly via cartilage intermediate (endochondral ossification) (Hu et al., 2017). During endochondral ossification, VEGF binds to the cartilage matrix until its release by MMPs. MMPs degrade and remodel the extracellular matrix (ECM) and are highly expressed during fracture repair (Bahney et al., 2019; Table 1). Knockout of MMP-2 delays bone remodeling, while MMP-9 and MMP-13 knockouts impair cartilage remodeling, vascularization and bone formation (Colnot et al., 2003; Behonick et al., 2007; Wang X. et al., 2013). Interestingly, administration of recombinant VEGF rescues these phenotypes, emphasizing the importance for MMP mediated fracture revascularization of VEGF availability (Colnot et al., 2003; Bahney et al., 2019). ECM proteins such as thrombospondin and osteopontin also modulate fracture vascularization. Thrombospondin has an antiangiogenic function (Bahney et al., 2019). Accordingly, thrombospondin knockout mice exhibit enhanced angiogenesis and bone regeneration (Taylor et al., 2009; Miedel et al., 2013; Table 1). In contrast, osteopontin is a proangiogenic factor, and delays fracture neovascularization when deficient (Duvall et al., 2007; Bahney et al., 2019; Table 1). During the remodeling phase, callus and vessels are reduced toward pre-injury levels, and the cortical and medullary structure is restored (Baker et al., 2018). The remodeling process consists of a complex interplay between osteoclasts, osteoblasts and vasculature and is driven by high levels of proinflammatory cytokines such as IL-1 and TNF-α (Mountziaris and Mikos, 2008; Baker et al., 2018). Blockade of angiogenesis during this phase significantly increases callus formation and inhibits callus remodeling (Holstein et al., 2013).

During the remodeling phase, BM stem cells (BMSCs) form a source of osteoclasts, while a subset of periosteal stem cells differentiates into chondrocytes and osteoblasts (Colnot, 2009; Baker et al., 2018). Moreover, BMSCs and pericytes direct stem cell differentiation by producing various trophic factors (Colnot, 2009; Hadjiargyrou and O’Keefe, 2014; Baker et al., 2018). Multiple studies have indicated an MSC-function for pericytes, enabling them to differentiate into osteoblast and chondrocyte progenitors (Diaz-Flores et al., 1992; Maes et al., 2010; Figure 3).

Comorbidities such as aging significantly delay fracture repair, presumably due to underlying vascular dysfunction (Bahney et al., 2019). Blood vessel density was significantly decreased in fractures of aged and middle-aged mice compared to young mice, coupled with reduced cartilage volume (Lu et al., 2008). Moreover, expression levels of VEGF, HIF-1α, MMP-9, and MMP-13 were significantly reduced in early fracture calluses of aged mice, likely underlying the observed delay of angiogenesis (Lu et al., 2008).

## Dysregulation of Bone Vasculature in Bone Malignancies

The BM provides a unique microenvironment not only for HSCs but also for tumor cells. Similar to HSCs, cancer stem cell (CSC) activity relies on signals from the BM microenvironment (Plaks et al., 2015; Batlle and Clevers, 2017). Primary tumor cells and host cells secrete various factors that support CSC survival and dissemination, creating a pre-metastatic microenvironment (Kaplan et al., 2005; Chin and Wang, 2016; Sivan et al., 2019). Further, CSCs can modulate angiogenesis by producing proangiogenic factors such as VEGFA (Chand et al., 2016) to induce unregulated tumor angiogenesis and metastasis (Carmeliet and Jain, 2011; Treps et al., 2017; Sun et al., 2020; Figure 3 and Table 1). Here, malignant alterations of the BM niche in hematologic tumors, primary bone tumors and bone metastasis are highlighted.

### Hematologic Tumors

Acute myeloid leukemia (AML) is the most common type of leukemia. AML patients show upregulated VEGF levels and BM hypervascularity, associated with poor prognosis (Bosse et al., 2016). Besides inducing tumor angiogenesis, VEGFA also facilitates chemotactic cell migration and increases vascular permeability (Nagy et al., 2008; Heinolainen et al., 2017). Studies using intravital two-photon microscopy have demonstrated various structural and functional maladaptations of bone vasculature in AML. Vasculature of mice with AML show disorganized vasculature, reduced vessel diameter and increased microvascular density within the BM and reduced vessel density in the endosteal region (Passaro et al., 2017; Duarte et al., 2018). Also, AML mice exhibit functional vascular abnormalities; perfusion is impaired, while angiogenic VEGFA levels, hypoxia and vascular leakiness are increased (Passaro et al., 2017; Duarte et al., 2018; Figure 3). These findings are supported by the transcriptomic analysis of ECs after human AML engraftment, revealing reduced endothelial expression of tight junction components that are required for vessel integrity (Passaro et al., 2017). Xenografts of AML patients show an increase in perivascular hypoxia that increases endothelial ROS and NO levels, impairs HSC function and promotes cell death and HSC egress from the BM (Passaro et al., 2017). Inhibition of endothelial remodeling in AML rescues HSC loss and increases chemotherapeutic efficiency and survival (Duarte et al., 2018).

### Primary Bone Tumors

Osteosarcoma is the most common primary bone tumor (Broadhead et al., 2011a). It is considered highly vascularized and is characterized by early metastatic dissemination through intratumoral vessels. The lungs and bone represent the most common sites of metastasis (Broadhead et al., 2011a; Kunz et al., 2015). Microvascular density analysis of osteosarcoma patient biopsies revealed increased survival rates and responsiveness to chemotherapy in patients with low osteosarcoma vascularization (Kunz et al., 2015). Similar to other blood and bone cancers, osteosarcoma cells have a strong angiogenesis-inducing function that increases with intratumoral vessel size and length (Uehara et al., 2014). Tumor angiogenesis is facilitated by a hypoxic and acidic microenvironment around proliferating osteosarcoma cells, which stimulates HIF-1α and subsequent VEGF upregulation (Broadhead et al., 2011a). Antiangiogenic factors and proteins, including thrombospondin-1, TGF-β (Ren et al., 2006) and pigment epithelial-derived factor (PEDF) (Cai et al., 2006) are downregulated in osteosarcoma. PEDF has shown promising results as an anti-tumor agent (Table 1). Multiple studies have demonstrated suppression of tumor growth, angiogenesis and metastasis upon overexpression (Ek et al., 2007b) and systemic administration of PEDF *in vivo* and *in vitro* (Ek et al., 2007a; Broadhead et al., 2011b).

Upregulation of the proangiogenic protein CYR61 in osteosarcoma, crucially contributes to primary tumor vascularization (Habel et al., 2015; Table 1). Silencing CYR61 decelerates tumor growth and reduces tumor vasculature and the expression of proangiogenic factors, including VEGF, PECAM and angiopoietins. Simultaneously, silencing of CYR61 upregulates thrombospondin-1 and other antiangiogenic factors (Habel et al., 2015; Table 1). Interestingly, CYR61 downregulation is associated with decreased MMP2 expression, an essential regulator of metastatic osteosarcoma capacity (Habel et al., 2015). MMPs play an important role in degrading the ECM to enable tumor invasion into the surrounding tissue (Oh et al., 2001; Broadhead et al., 2011a). Membrane-type 1 matrix metalloproteinase (MT1-MMP) is crucial for cell migration and has been shown to promote tumor cell migration and invasion (Itoh et al., 2001; Itoh, 2006). They also remodel the vascular network and decrease vessel wall integrity to allow tumor cell passage into the bloodstream (Oh et al., 2001; Broadhead et al., 2011a) and stimulate tumor angiogenesis via releasing ECM-bound VEGF (Bergers et al., 2000).

Tumor cells further express NGF, which has similar angiogenic effects as VEGFA (Nico et al., 2008). NGF induces tumor angiogenesis by enhancing endothelial growth, migration and permeability (Romon et al., 2010). Inhibition of NGF with siRNA significantly reduces tumor progression and angiogenesis in breast cancer (Adriaenssens et al., 2008), while overexpressing TrkA increases tumor development and angiogenesis (Lagadec et al., 2009). Both VEGFA and NGF promote MMP production (Pufe et al., 2004; Shan et al., 2013) facilitating tumor invasion and cancer angiogenesis (Belotti et al., 2003; Okada et al., 2004).

Interestingly, cancer cells integrate into the vasculature and fuse with ECs, to contribute to the tumor vasculature, acquiring an EC-like phenotype, a process termed as “vascular mimicry” (Maniotis et al., 1999; Cogle et al., 2014). *Trans*-differentiation of CSCs into pericytes is reported to occur via endothelial production of CXCL12 and TGF-β (Cheng et al., 2013; Figure 3 and Table 1). The acidic tumor microenvironment increases CXCL12 production (Nakanishi et al., 2016), stimulates angiogenesis, tumor proliferation and chemoresistance (Meng et al., 2018). Vascular mimicry has been described in numerous types of solid tumors, including osteosarcoma and Ewing sarcoma and is involved in cancer progression, dissemination and metastasis (Ge and Luo, 2018).

### Bone Metastasis

The BM vascular niche acts as a protective and supportive site for cancer cells (Ninomiya et al., 2007). BM hematopoietic cells express VEGFR1, thereby forming a pre-metastatic niche that attracts cancer cells (Kaplan et al., 2005; Table 1). Due to its intrinsically high vascular density, the BM enables increased crosstalk between cancer cells and ECs, supporting tumor cell proliferation (Virk and Lieberman, 2007).

Endothelial thrombospondin-1 production creates a stable BM vascular niche for disseminated tumor cells (DTCs). Integrated into this niche, DTCs remain in a dormant state over a long period (Ghajar et al., 2013; Kusumbe, 2016; Qu et al., 2019). The large vessel diameter and low sinusoidal blood flow of the BM vasculature facilitate DTC dormancy and therapy resistance (Kopp et al., 2005; Kusumbe, 2016). Co-culture of AML cells with BM ECs increases the proportions of quiescent AML cells (Cogle et al., 2014). Integrin-mediated interaction between DTCs and endothelial-derived von Willebrand factor and VCAM1 is a crucial factor in DTC chemoresistance (Carlson et al., 2019). Inhibiting these interactions via integrin-blocking antibodies sensitizes DTCs to chemotherapy and prevented bone metastasis (Carlson et al., 2019).

Endothelial PDGF-B signaling is another key regulator of tumor cell dormancy and therapy resistance (Singh et al., 2019; Table 1). Radiation and chemotherapy induces a bone-specific expansion of pericytes via endothelial PDGF-B signaling. Expanding pericytes further support therapy resistance of quiescent DTCs in the BM by secreting quiescence-inducing factors such as CXCL12 (Singh et al., 2019). DTC dormancy is guided by microenvironmental cues similar to those involved in adult stem cell dormancy (Risson et al., 2020). For instance, MSC-specific deletion of CXCL12 promotes LSC division and expansion while reducing normal HSC numbers (Agarwal et al., 2019).

Remodeling of the tumor BM microenvironment such as the age-related loss of perivascular PDGF-B signaling reactivates dormant DTCs and induces their proliferation (Ghajar et al., 2013; Singh et al., 2019). Therefore, bone is one of the most common sites of metastasis even after decades of latency (Kusumbe, 2016; Singh et al., 2019). Quiescent DTCs retain transcriptional plasticity that enables them to reactivate different regulatory programs, allowing reversible growth arrest and survival (Risson et al., 2020). Induction of cell cycle expression in AML cells renders them susceptible to therapy, leading to tumor regression (Bosse et al., 2016). Moreover, inhibiting VEGFA signaling improves chemotherapy efficiency (Poulos et al., 2014).

Furthermore, bone metastasis is the most common cause of pain in cancer (Mercadante, 1997). NGF plays an important role in modulating bone cancer pain and is expressed by tumor, immune and inflammatory cells (Dollé et al., 2003; Sevcik et al., 2005; Table 1). Treatment with an anti-NGF antibody significantly reduces bone cancer pain behaviors in a mouse model of femoral osteosarcoma (Sevcik et al., 2005). The NGF monoclonal antibody tanezumab has also shown promising results in the treatment of chronic pain in patients with metastatic bone cancer (Sopata et al., 2015).

## Radiation and Chemotherapy

Radiation is a commonly used therapy for hematological malignancies, that causes tissue damage, reduces hematopoietic populations and promotes HSC mutations. Despite its common prescription, the effects of radiation therapy on the BM microenvironment remain poorly understood. Microcomputed tomography and immunohistological studies of irradiated murine bone show a severe decline of bone volume with an increase in number and activity of bone-resorbing osteoclasts (Willey et al., 2008; Kondo et al., 2009; Batsivari et al., 2020). Radiation also damages the BM vasculature, particularly depleting sinusoidal ECs and increasing vascular dilation and permeability. Transcriptomic analysis revealed substantial changes in EC transcriptomes in response to radiation, including genes associated with vascular niche function (Chen et al., 2019). Furthermore, expansion of Apln-expressing ECs occurs upon radiation treatment. This subpopulation of bone ECs has the ability to generate arterial ECs and contribute to BM vascular regeneration after irradiation (Chen et al., 2019) and represents a subset of type H ECs. Moreover, radiation significantly enhances the BM adipocyte compartment (Hooper et al., 2009; Zhou et al., 2017; Figure 1). Regeneration of sinusoidal ECs post-irradiation is partially mediated by VEGFR2 signaling and is essential for the restoration of normal hematopoiesis (Hooper et al., 2009; Table 1).

Another commonly used therapy for hematological malignancies is chemotherapy. Most chemotherapies have similar effects on the BM microenvironment as radiation, depleting osteoblasts, increasing adipocyte numbers and damaging BM endothelium (Zhou et al., 2017; Batsivari et al., 2020). Tracking gene expression profiles of BM cells after 5-FU treatment revealed an upregulation of adipogenesis-associated genes accompanied by a downregulation of osteolineage-associated genes. Chemotherapy is further associated with a general loss of vascular and perivascular cells in the BM (Tikhonova et al., 2019). Myeloablation via radiation or chemotherapy increases expression of inflammatory cytokines such as IL-6, G-CSF, and GM-CSF that promote HSC differentiation and lineage commitment (Rafii et al., 2016). In contrast, myeloablation downregulates vascular Notch delta ligands, Dll1 and Dll4. Dll4 is a regulator of hematopoietic differentiation, causing transcriptional reprogramming and myeloid priming of HSCs in its absence (Tikhonova et al., 2019; Table 1). After myeloablation, ECs upregulate their production of VEGFA, FGF-2, and other angiogenic factors to facilitate HSC regeneration. These angiocrine factors activate Akt and upregulate the Notch ligand Jagged-1 (Kobayashi et al., 2010), suggesting a key role of Notch activation in HSC regeneration. Transplantation of Akt-activated BM ECs enhances hematopoietic recovery after myeloablation (Poulos et al., 2015). Further, co-activation of endothelial Akt with MAPK induces HSC differentiation and expands the hematopoietic progenitor pool, demonstrating a key role of Akt in hematopoietic regeneration (Kobayashi et al., 2010; Rafii et al., 2016). Collectively, these findings demonstrate significant BM vascular niche remodeling during hematological malignancies, myeloablation and hematopoietic recovery.

## Concluding Remarks

Bone marrow ECs and perivascular cells form a heterogeneous and nurturing microenvironment for stem and progenitor cells of various lineages and produce various factors to support hematopoiesis and osteogenesis. Aging, inflammation and other stress factors can alter vascular morphology and function and disrupt angiocrine crosstalk in vascular niches. Pathological processes including arthritis, osteoporosis, bone pain and cancer are associated with bone angiogenesis. Vascular niche remodeling in response to stress can severely affect HSCs, hematopoiesis and bone lineage cells and may contribute to metastatic relapse and chemoresistance. Detailed knowledge of the BM microenvironment could provide new insights into pathological processes in the skeletal system and holds the potential to provide strategies for the clinical management of hematological disorders and bone diseases. Thus, future research should further unravel the impact of stress on the BM vascular niche to improve our understanding of vascular niche function and interactions.

## Author Contributions

SS wrote the original draft. SS and JC prepared the figures. AK, JC, and FW reviewed and edited the manuscript. AK designed the review structure and figures. All authors contributed to the article and approved the submitted version.

## Conflict of Interest

The authors declare that the research was conducted in the absence of any commercial or financial relationships that could be construed as a potential conflict of interest.
